# Vascular plant abundance and diversity in an alpine heath under observed and simulated global change

**DOI:** 10.1038/srep10197

**Published:** 2015-05-07

**Authors:** Juha M. Alatalo, Chelsea J. Little, Annika K. Jägerbrand, Ulf Molau

**Affiliations:** 1 Department of Ecology and Genetics, Uppsala University, Campus Gotland, 621 67 Visby, Sweden; 2 VTI, Swedish National Road and Transport Research Institute, Box 55685, 102 15 Stockholm, Sweden; 3 Department of Biological and Environmental Sciences, University of Gothenburg, PO Box 461, 405 30 Gothenburg, Sweden

## Abstract

Global change is predicted to cause shifts in species distributions and biodiversity in arctic tundra. We applied factorial warming and nutrient manipulation to a nutrient and species poor alpine/arctic heath community for seven years. Vascular plant abundance in control plots increased by 31%. There were also notable changes in cover in the nutrient and combined nutrient and warming treatments, with deciduous and evergreen shrubs declining, grasses overgrowing these plots. Sedge abundance initially increased significantly with nutrient amendment and then declined, going below initial values in the combined nutrient and warming treatment. Nutrient addition resulted in a change in dominance hierarchy from deciduous shrubs to grasses. We found significant declines in vascular plant diversity and evenness in the warming treatment and a decline in diversity in the combined warming and nutrient addition treatment, while nutrient addition caused a decline in species richness. The results give some experimental support that species poor plant communities with low diversity may be more vulnerable to loss of species diversity than communities with higher initial diversity. The projected increase in nutrient deposition and warming may therefore have negative impacts on ecosystem processes, functioning and services due to loss of species diversity in an already impoverished environment.

The Earth’s ecosystems are under ever-increasing pressure of global change due to anthropogenic activities. In addition to the direct pressure of land use change where natural ecosystems are altered by human activities (e.g. farming, clear cutting and the spread of human infrastructure), indirect pressure is growing due to increased deposits of nutrients^1^,^2^ and changing climate due to increased green house gas emissions^3^. These global changes will likely have large impacts on ecosystem processes^4^.

The effects of global change have already been measured in many systems. For instance, increased nitrogen (N) deposits in the UK, Europe, and the United States have already had some visible negative impact on ecosystems, including leading to a decline in species richness and impacts on plant community composition^2^,^5^. However, nutrient addition does not always cause negative impact on species richness and diversity. A study across spatial scales, using data from 18 experiments across North America with N enrichment, revealed contrasting results regarding the impact on β diversity. N addition was found to have a positive effect on β diversity in low productivity sites, while it decreased β diversity in high productivity sites^6^. Also, communities have been shown to recover slowly from the changes caused by nutrient addition^7^. Global change can affect interspecific interactions, including interactions between plants and animals^8^. As in many fields of ecology, the majority of available prior research is not distributed evenly across taxa or geography; in most cases the studies originate from Europe and North America^9^.

In arctic and alpine systems in particular, nutrient deposition decreases the tundra’s capacity for storing and cycling of carbon^10^ and nitrogen^11^; hence, global change has profound implications for arctic ecosystem functioning. Indeed, alpine and arctic tundra already exhibits evidence of recent changes in plant community composition, biomass and diversity^12^,^13^. This is believed to be in part a response to the increase in temperature that has occurred in the Arctic during the past 40 years, a warming trend reaching on average 0.4 °C per decade, much higher than on lower latitudes^14^. Changes in vegetation have also been detected across a wide array of mountain summits in Europe, with upward shift of plant species but contrasting effects on species richness^15^,^16^, however a northward movement can not always be linked to climate change^17^. The increase in temperature is predicted to persist in the future, with warming continuing to develop fastest in the polar regions^3^. And this may affect vegetative growth, reproduction and phenology of plant in severe environments^18^,^19^,^20^.

The combination of enhanced warming and nutrient deposition can have a particularly high impact on nutrient-limited ecosystems^10^,^21^. Heaths, high alpine areas, and arctic tundra are among those areas that are most likely to be sensitive to the changes in ambient temperature and nutrient availability^22^. Experimental studies on global change in cold regions have revealed that there is high variability in responses to nutrient addition and warming. For instance, responses may vary between and within plant functional groups: evergreen shrubs in Arctic and alpine areas have been affected by nutrient addition negatively, positively, and neutrally^23^,^24^,^25^,^26^. Forbs exhibit contrasting responses to nutrient addition in experiments simulating global change^21^,^27^, while graminoids have to a larger extent shown consistent responses to nutrient addition^26^,^27^,^28^,^29^, but not always^30^. As a result, short and medium term experiments indicate that global change may cause shifts in the hierarchical structure of plant communities^30^. There is also temporal variation in responses, as shrubs, cushion plants, and whole communities for instance, have shown differing short- and longer-term effects of nutrient addition^20^,^26^,^30^, whereas total communities may show contrasting responses to warming^31^. Finally, the initial conditions of the communities may determine response to global change, as well as biotic interactions^32^.

In this study, we examine whether initial short-term responses of vascular plants in a sub-arctic alpine heath to a factorial warming and nutrient addition are consistent moving into the medium-term. Dry heath sites are characterized by lichens which often decrease with perturbations^28^,^31^, while lichens constitute a less substantial portion of more moist communities. Analysis of this site after three^28^ and five years^33^ of manipulation showed substantial changes to community structure; we ask whether these short-term responses to simulated global change are good predictors of longer-term responses in heaths. In a similar experiment in a nearby rich meadow, responses were non-linear over time and the short-term responses were shown to be poor predictors of medium-term responses^30^. Here we studied the development of dominance hierarchies, species richness and diversity of vascular plants in a heath community. Specifically we hypothesized that 1) dominance hierarchies would shift in response to experimental nutrient addition; and 2) species richness and diversity of vascular plants would increase in response to the nutrient addition and the combined warming and nutrient addition.

## Results

### Observed ambient changes

**Figure 1:**
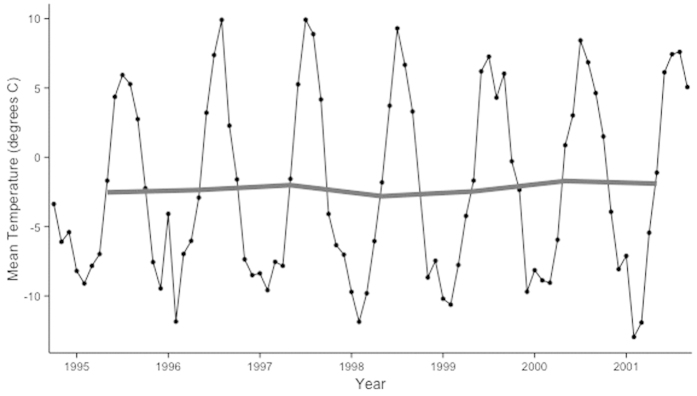
Annual and monthly mean temperatures. Monthly mean temperatures over the course of the study period (black line) and yearly mean temperatures (gray line), which varied by just over one degree Celsius in seven growing seasons.

Mean annual temperatures ranged from –1.71 to –2.81 °C from the autumn of 1994 to autumn 2001, with a 20 °C difference in mean temperature between the winter and summer months (Fig. 1). Total annual precipitation ranged between 682 and 1069 mm. In 1997, the year with the largest number of growing degree days, growing degree days amounted to 389, compared to 183 in 1995 (Table 1).

**Figure 2:**
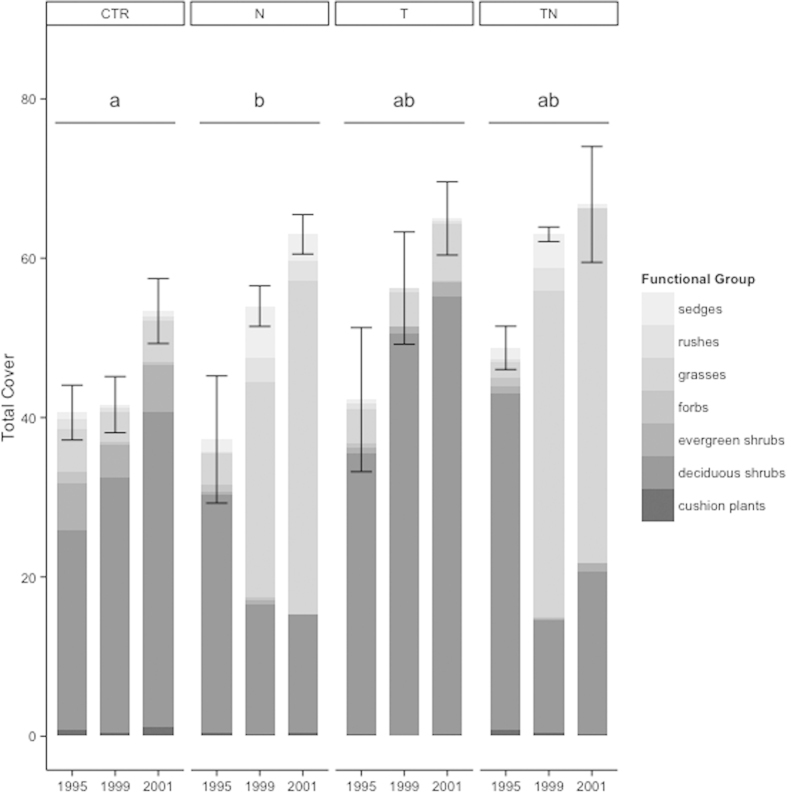
Total cover and proportional functional group cover by year. Total cover in experimental plots in 1995, 1999, and 2001, with the proportion of this cover made up of seven different vascular plant functional groups. Error bars represent one standard error of the total cover. Treatments with the same letter marking showed no difference in total cover according to Tukey’s HSD tests, while those with different letters were significantly different (p < 0.05). In this and all subsequent figures, CTR = control plots, N=nutrient enhancement treatment, T = warming treatment, and TN = combined nutrient and warming treatment.

Between 1995 and 2001, we found a significant increase in total cover of control plots (t = 2.259, p = 0.03), equating to a 31% rise. The majority of plant cover increase being between 1999 and 2001. This was concurrent with a nonsignificant trend of increasing cover across the entire experiment over this time period, encompassing both the control and treatment plots (Fig. 2). However, there were no significant changes in species richness or diversity (Table 2).

### Changes in response to perturbations

Total vascular plant cover varied significantly with both warming (linear mixed-effects model, F_1,55_ = 9.36, p = 0.003) and nutrient addition (lme, F_1,55_ = 6.40, p = 0.01). However, when treatments were compared in a pairwise fashion, only the nutrient addition increased total cover compared to control plots (Tukey HSD, p = 0.002, Fig. 2).

**Figure 3:**
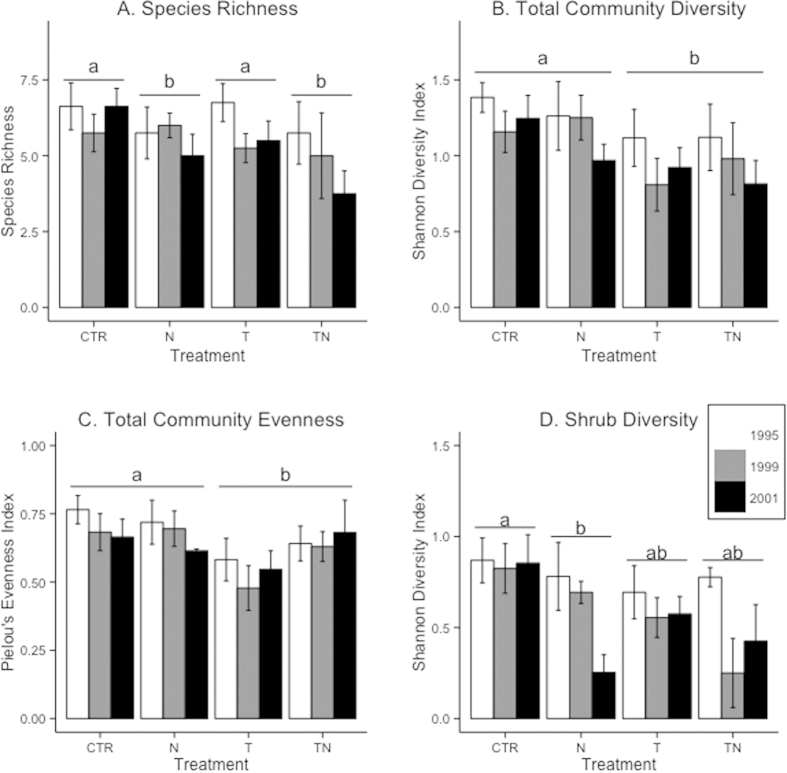
Total community diversity and evenness among years. Total species richness (**A**), community diversity as measured by the Shannon diversity index (**B**) and Pielou’s evenness index (**C**) in the experimental plots in 1995, 1999, and 2001. Treatments with the same letter marking showed no difference in Tukey’s HSD tests, while those with different letters were significantly different (p < 0.05). Shrub diversity (**D**) responded to both temperature and nutrient manipulation (linear mixed-effects model, p < 0.02).

Across the seven-year experiment, 16 species of vascular plants were found in the heath community, comprising one cushion species (*D. lapponica*), four deciduous shrubs, four evergreen shrubs, two forbs, two grasses, and three sedges. Nutrient addition had a significant negative effect on species richness (lme, F_1,55_ = 4.55, p = 0.04; Fig. 3A). There was no significant interaction effect (LRT, p > 0.5) between nutrient and warming for either total cover or species richness. Shannon’s diversity (lme, F_1,55_ = 7.71, p = 0.008) and Pielou’s evenness (lme, F_1,55_ = 6.50, p = 0.02) of the vascular plant community showed a significant responses to the warming treatment. For Shannon’s diversity, the warming and combined treatments of warming and nutrient addition showed significantly lower diversity than the control or nutrient addition (Tukey HSD, p = 0.006; Fig. 3B). Evenness was lower in treatments with warming compared to control and nutrient-only plots (Tukey HSD, p = 0.01; Fig. 3C). Shrub diversity showed significant effects of both the nutrient and warming treatments (lme, nutrient: F_1,55_ = 7.33, p = 0.009; temperature: F_1,55_ = 3.94, p = 0.05) but no interaction between the two. From the pairwise comparisons between treatments, only the control and nutrient addition treatments differed significantly from one another (Tukey HSD, p = 0.004; Fig. 3D). Diversity of graminoids did not respond to either nutrient addition or warming (linear mixed-effects model, p > 0.2).

**Figure 4:**
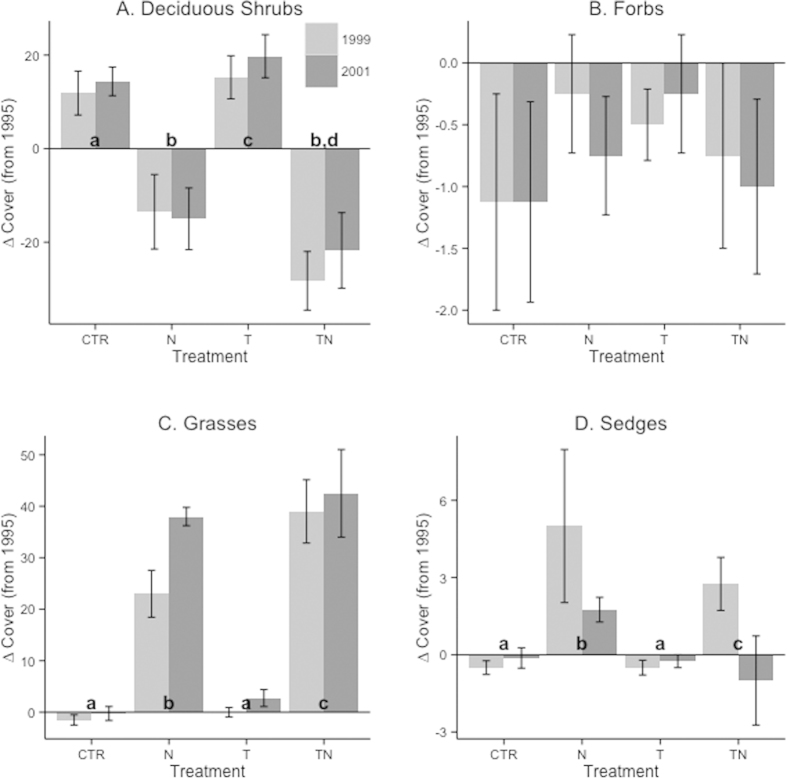
Change in cover of functional plant groups. Absolute change in cover from 1995 to 1999 (light grey bars) and 2001 (dark grey bars) for deciduous shrubs (**A**), forbs (**B**), grasses (**C**), and sedges (**D**). Where significant main effects were detected, Tukey’s HSD tests were applied to determine whether there were significant differences among treatments (p < 0.05). Error bars represent one standard error.

The significant increase in total cover over the course of the experiment was the sum of both positive and negative changes in cover among the functional groups. Deciduous shrub cover responded to a significant nutrient by temperature interaction (lme, F_1,35_ = 3.80, p = 0.05; Fig. 4A). Deciduous shrub cover increased by similar amounts in the control and temperature plots by 1999 and maintained this increase through 2001; however in the treatments with nutrient addition, deciduous shrub cover decreased by as great or even a greater extent. This corresponded to a reduction to about 50% of their original cover (Fig. 2). Forbs and evergreen shrubs showed no significant effects of either warming or nutrient amendment (lme, p > 0.3), though forb cover showed a consistent decline over the course of seven years in all treatments including the control (Fig. 4B). Grass cover responded to both the warming (lme, F_1,36_ = 4.86, p = 0.03) and nutrient (lme, F_1,36_ = 198.09, p < 0.001) treatments, with significant differences between almost every pair of treatment types (Tukey’s HSD, p < 0.04 for all pair except control-temperature; Fig. 4C). This corresponded to grasses becoming by far the dominant functional group in the nutrient and combined temperature and nutrient treatments by 2001 (Fig. 2). Finally, sedge cover showed a significant response to the nutrient addition (lme, F_1,36_ = 9.44, p = 0.004; Fig. 4D). Sedge cover first expanded to more than four times its original extent in the nutrient plots in 1999, then decreasing slightly to only twice its original extent in 2001. In the combined nutrient and temperature treatment, these contrasting short- and medium-term responses were also apparent, with sedge cover increasing to double its original extent by 1999, but then dropping to just one third of its original extent by 2001 (Fig. 2). The rush and cushion plant functional groups did not have sufficient abundance (mean cover <1 for three or more treatment types) to make analyses robust; cushion plants were most common in control plots and rushes in the nutrient and combined temperature and nutrient plots (Fig. 2). On the whole, these changes in functional group cover led to drastic effects on dominance hierarchy, with grasses to large extent replacing deciduous shrubs in the nutrient and combined nutrient and temperature treatments.

## Discussion

In addition to the here, and elsewhere^12^,^13^, documented impacts of current climate change on vegetation, we found additional significant effects of nutrient addition and warming. Most evidently, this was seen as a shift in community hierarchy from a deciduous shrub-dominated system to a grass- and sedge-dominated system when nutrients were added. Previous short-term^33^ and long-term studies^26^ on alpine and arctic heaths have reported that nutrient addition causes increase of graminoids. We found this to be true over both the short- and medium-term (five and seven years), and that this pattern governed changes in diversity and community structure. However, graminoids do not respond equally “as a group”. Sedges that initially increased their abundance dramatically to the five years of nutrient addition and the combined nutrient addition and warming, had decreased their abundance after seven years compared to the five-year response. This was not the case for grasses, which continued to increase their dominance over the course of the entire experiment in response to the nutrient addition and the combined nutrient addition and warming. Nutrient perturbation seems more important than temperature perturbation in this nutrient- and species-poor heath system. There have been comparable findings in other studies on heaths, tussock tundra and grasslands^21^,^24^,^34^. This increase in plant growth in response to nutrient addition has been suggested to be due to widespread nutrient limitation in many ecosystems^21^. This idea is supported by data from the Alaskan Arctic showing that nine years of nutrient addition in tussock tundra increased the nutrient pool in all growth forms in the vegetation community^21^. The change in community structure is thought to be driven by the fact that graminoids, which have higher turnover rates, can exploit the increased nutrient levels much more rapidly than shrubs^35^. Results from a long-term experiment (22 years) indicate that this community shift from shrubs to graminoids under nutrient addition could be sustained over the longer term^26^.

Meanwhile, the effect of long-term warming differs among experimental sites, from no effect on above ground growth to 22 years of warming in a subarctic heath, to a strong positive effect of 20 years of warming on woody plants in Alaskan Tussock Tundra^26^,^36^. Observational and remote sensing studies have shown a steady shrub expansion in the Arctic^37^, assumed to be a response to warming. We suggest that nutrient perturbation has a stronger effect on shrub abundance. While not experiencing any ambient warming, similar to the documented trend of increasing shrub encroachment in tundra systems, we found 56% increases in deciduous shrub cover over the seven-year time scale. However, nutrient addition has an opposing effect and overrode the positive response to warming when we combined the two treatments. As discussed previously, nutrient perturbations have been shown to have effects on shrubs ranging from positive to negative or sometimes neutral. We found strong evidence that nutrient amendment has negative effects on deciduous shrub abundance in the species-poor alpine heath. The initial short-term (five years) response was not compensated for in the longer term (seven years). After seven years deciduous shrub cover was still significantly lower than it had been before the perturbations began; however, it did not further decline, either. Thus, we found nutrient perturbation to be a stronger driver of change than warming was, with the net result of a regime shift towards a grass dominated community rather than the shrub encroachment typified by warming. Future levels of nutrient deposition should be taken into account when predicting whether shrub encroachment will continue. In addition, nutrient and species poor heaths represents a unique type of ecosystem, and our findings highlight the fact that changes cannot be assumed to be homogenous across the many biotic and physical conditions which are lumped together under the umbrella of “tundra” ecosystems. Indeed, effects are likely to be variable across both space and time^38^.

This concept also underlies the fact that we found a negative effect of nutrient addition on species richness over the seven years of perturbations. Both the warming and the combined nutrient addition and warming perturbations negatively impacted the Shannon diversity and Pielou’s evenness indices, and brought a significant decline compared to the control and nutrient addition plots. This was counter to our hypothesized increase of diversity in response to the nutrient addition and the combined warming and nutrient addition, which was based on a similar study at a high Arctic site which found an increase in species richness in response to nutrient addition^23^. Thus, we had expected recruitment of new species in our experimental plots. The nutrient and species poor heath had neither a closed canopy nor a tough bottom layer to start with, and thus should have allowed opportunity for new recruitment if environmental conditions improved (more nutrients and/or warmer microclimate). Also, the valley where the heath is located has a number of diverse plant communities within a short distance enabling seed dispersal from new species, and the heath community actually experiences a more species-rich seed rain than is reflected by existing vegetation^39^. Increased nutrient levels have been found to decrease species richness as the communities became more competitive and developed more dense canopies increasing competition for light^28^. Indeed, alpine plants have been shown to decrease in biomass when light availability is experimentally attenuated^21^. Many native species may be poor competitors in these “improved” conditions, and instead fill their current niches because they are well-adapted to harsher conditions; when fertility and productivity increase, they may be displaced by the competition^40^. Open Top Chambers (OTCs) may also have functioned as physical barriers for recruitment, but this should not have been the case for the nutrient addition plots.

This decline in species richness and diversity has been reported in response to increased nutrient levels from several studies in a broad range of ecosystems^5^,^41^,^42^, but not always^30^. The decline in species diversity is a reason for concern as there is evidence that decline in species diversity may affect ecosystem processes, functioning and services^43^,^44^,^45^. A study on 17 biodiversity experiments revealed that 84% of grassland plant species in the experiments promoted ecosystem functioning^46^. Furthermore, the importance of the different species was shown to vary among years, space, functions and different environmental change scenarios, making it difficult to predict which species are most important for ecosystem services^46^. The interplay of dominance between the evergreen shrub *Empetrum nigrum*, a congener of the *Empetrum hemaphroditum* species found in our dataset, and the deciduous shrub *Betula nana*, which was among the dominant species in our dataset, has been shown in millenial-scale models to have important effects and feedbacks on ecosystem processes^47^.

Long-term assessments of unmanipulated arctic and subarctic heath communities have found significant changes in species richness and diversity, ranging from positive^48^ to neutral^49^ and negative^31^,^41^. Similar to a study in the Canadian High Arctic^49^ we found no significant changes in richness or diversity in the control plots, neither did we observe any ambient warming during the period of the study. A possible explanation could be that the nutrient and species poor heath is a rather “extreme” environment where vascular plants have difficulty establishing. Several of the studies which found strong effects of ambient warming included lichens, a dominant and diverse functional group in arctic heath. It is possible that if we had also included lichens effects on richness and diversity would have been more pronounced, as lichen abundance was shown to increase, though not significantly so, in the control plots in the first five years of the experiment^33^.

We did, however, find other responses in the control plots. Vascular plant abundance in the heath increased over the course of the seven-year experiment in all treatments, with the control plots’ abundance significantly increasing by 31% over this time period. This increase is in line with a nine-year study in a subarctic heath in a nearby valley, which found increased aboveground biomass in control plots after nine years between 1991–1999^50^. More broadly, a meta-analysis of control plots in 158 experimental warming studies from 46 sites (ranging from 1980 to 2010) found that height of shrubs, graminoids, and forbs and abundance of shrubs increased during ambient warming^51^. However, not all studies have found an increase of plant growth over this timescale, with one 20-year study in northern Sweden finding no change in vascular plant cover despite 2 °C of warming over the study period^41^.

In the majority of the observational studies which have found increasing biomass and/or abundance, the changes have been attributed to warming already occurring in the system. However, we did not observe any clear increase of temperature during the period of the study, therefore it is more likely that the observed increase in abundance in the nutrient and species poor heath may be due to variation in growing degrees days and mean temperature among years. The 1994–1995 growing season, which was used as the baseline and initial sampling season, had the shortest growing season of any of the seven years during our study, when measured as growing degree days. Meanwhile, 2001 had substantially higher number of growing degree days than 1995 and 1999. Furthermore, while mean temperature varied among years, the mean temperature of 2001 was roughly 0.6 °C warmer than 1995 and 1999. The variation in growing degree days and mean temperature could therefore partly explain the observed increase in cover. Over the longer term, the region in Northern Sweden where our study site is located has experienced annual warming of 0.2° per decade and December-January-February warming of 0.5° per decade from 1956 to 2004^52^. This increase in temperature may promote plant growth, as it has in other arctic and subarctic studies^49^. Increase in abundance and biomass in the Alaskan arctic has been suggested to be caused due to higher mineralization rates in the soil at warmer temperatures^21^. Given our experimental findings that nutrient amendment has a far more substantial effect on vascular plant abundance than does warming, an increased mineralization over longer term due to ambient warming could cause delayed growth responses and shifts on dominance hierarchies. However, winter climate changes may also be important. Annual precipitation has significantly increased by 1.3 cm per decade in Abisko area from 1914 to 2004, which corresponded to an increase in December-January-February snow depth of 1.7 cm per decade; at our site in particular, the 1999–2000 winter and growing season had particularly high precipitation. Snow manipulation experiments have shown that increased winter snow cover insulates tundra plants from freezing events and increases spring runoff, and that vascular plant cover and biomass may increase as a response^53^.

In terms of conservation applications the total community should be protected in order to ensure that the most important species are included. Plant communities with higher biodiversity have been shown to be more efficient and more stable, which may prevent strong declines in ecosystem functioning after perturbation, and increase community resilience in communities with higher biodiversity^46^,^54^. A similar study in the same valley, but in a more species rich meadow, found drastic shift in dominance hierarchies but no effects on vascular plant diversity, suggesting that more diverse plant communities may be more stable compared to the species-poor heath^30^. Thus, our results indicate that low-biodiversity ecosystems such as alpine and arctic heaths are likely to become increasingly vulnerable to loss in species diversity in a future with higher ambient nutrient and temperature levels. The projected increase in nutrient deposition and warming may therefore have negative impacts on ecosystem processes, functioning and services due to loss of species diversity in an already impoverished environment.

## Methods

### Study area

The experimental site is located at the Latnjajaure Field Station (LFS) in northern Sweden, at 1000 m elevation in the valley of Latnjavagge (68°21´N, 18°29´E). Climate in the area can be classified as sub-arctic, having cool summers, relatively mild, snow-rich winters, the snow cover extending for most of the year. The valley is highly diverse with regard to physical conditions, from dry to wet and poor and acidic to base-rich, as reflected in its plant communities^28^,^30^. The present experiment focused on a heath community with low vascular plant diversity. No specific permits were needed for the described field studies, the land is not privately owned, not protected and the field studies did not involve endangered or protected species.

### Experimental design

In July 1995, 20 plots (1 × 1 m) with homogenous vegetation cover were chosen in the heath plant community and randomly assigned to treatments in a factorial design. There were 8 control (CTR) plots and 4 plots for each of the experimental treatments: warming (T for temperature enhancement), nutrient addition (N) and combined warming and nutrient addition (TN). Warming was induced by Open Top Chambers (OTCs) which increase temperature by 1.5 to 3 °C compared to control plots with ambient temperature^28^. Nutrient addition consisted of 5 g of nitrogen (as NH_4_NO_3_) and 5 g of phosphorus (P_2_O_5_) per m^2^, dissolved in 10 l of meltwater. In 1995 all plots were analysed with a point frame method (described below) to determine the species occurrences under natural conditions, prior to any implementation of experimental treatments, thereby achieving the BACI approach; Before-After-Control-Impact^55^. The OTCs were then left on plots with warming treatments year-around, and nutrient addition was applied directly after the initial vegetation analyses in 1995 and a few days after snow melt in the subsequent years (1996–2001). The nutrient treatments were then terminated after 2001.

### Measurements

All vascular plants in the plots were identified to the species level and cover of each species was assessed using a 1 × 1 m grid frame with 100 points, in the peak of the 1995, 1999, and 2001 growing seasons. Only the first hit was recorded. To ensure accuracy and reproducibility, the same grid frame was used for each measurement, and fixed points at the corner of each plot allowed the frame to be placed in the same position within the plot at each different measuring point. This method has been shown to be highly accurate for detecting changes in tundra vegetation^56^. For each plot at each time point, the total cover was calculated as the sum of point frame hits from all species. There were a varying number of point-frame gridpoints within the plot boundaries, so each plot was subsampled to have an equal sample size (83 points, the number of points in the smallest plot) using the ‘rrarify’ function in the R package ‘vegan’^57^. Total cover was also broken down by functional groups; deciduous shrubs, evergreen shrubs, cushion plants, forbs, grasses, and sedges. Grasses (i.e. *Poaceae*) and “sedges” (including rushes, i.e. *Cyperaceae* and *Juncaceae*) were separated from their usual classification together as graminoids because they have been shown to respond differently to fertilization both in a lichen-dominated heath and a meadow^27^,^30^. A single species of cushion plant, *Diapensia lapponica*, was present in some but not all plots and treatments, leaving an unbalanced design, and was thus not analysed as its own functional group. Species richness was counted for each plot at each occasion and used to calculate the Shannon-Weaver diversity index according to the R package ‘vegan’^57^. This diversity index was also calculated for broad functional groups of graminoids (grasses, sedges, and rushes combined) and shrubs (evergreen and deciduous combined); in more narrowly-defined functional groups we used to assess cover, species richness was often very low, i.e. a single species, making calculation of Shannon diversity impractical. Likewise, the forb functional group never comprised more than a single species present in a plot at any given time, making calculation of diversity inappropriate.

### Data analyses

To analyse the effect of existing global change on the alpine heath community, we used a priori orthogonal contrasts within a linear model to compare total cover, total species richness, and Shannon’s diversity in the eight control plots from 1995 to 1999 and 2001. Data were normally distributed and not heterogeneous; we tested for autocorrelation of plots using generalized least squares models^58^ with and without an explicit auto-regressive moving average (ARMA(1,0)) residual structure and comparing the two models using both AIC and likelihood ratio tests (LRT), with the result that the autocorrelation structure did not improve model fit (p > 0.5). These and all subsequent analyses were performed in R version 2.15.3 (R Core Team. *R: A language and environment for statistical computing*. R Foundation for Statistical Computing, 2013).

To assess the effects of experimental treatments over time on total cover, species richness, evenness, and Shannons diversity (total, graminoid, and shrub), we used a linear mixed-effects model. This was analysed using the R package ‘nlme’ with degrees of freedom calculated as described by Pinheiro and Bates^59^. Plot was nested within year as random effects, and nutrient and temperature enhancement were considered as interacting fixed effects. For each response variable, normality and homogeneity of variance were confirmed using standard diagnostic procedures. Significance of the nutrient by temperature interaction was tested by comparing nested models using maximum likelihood (ML). If the interaction was not significant, it was removed from the model. The best model was implemented using restricted maximum likelihood (REML). Treatment-level differences within significant fixed effects were examined using Tukey Honestly Significant Differences.

Cover was also analysed for the functional groups. Any plot where a functional group was not present in any of the three years was excluded from analysis (this applied to cushion plants, evergreen shrubs, forbs, and sedges; deciduous shrubs and grasses were present in every plot). For those plots where a functional group was present at least once, 1999 and 2001 cover was calculated as absolute change from the 1995 measurement. The relative change in total and functional group cover was analysed using the same mixed-effects model and posthoc testing as described above.

## Author Contributions

J.M.A. and U.M. designed the experiment. J.M.A., U.M. and A.K.J. and carried out fieldwork. C.J.L. carried out data analyses with direction from A.K.J., J.M.A. and C.J.L. drafted the manuscript. All authors read, commented and approved the final manuscript.

## Additional Information

**How to cite this article**: Alatalo, J. M. *et al*. Vascular plant abundance and diversity in an alpine heath under observed and simulated global change. *Sci. Rep.* **5**, 10197; doi: 10.1038/srep10197 (2015).

## Supplementary Material

Supporting Information Supplementary Dataset 1

## Figures and Tables

**Table 1:** Temperature and Precipitation in Latnjajaure.

**Growing season**	**Precipitation (mm)**	**Summer Maximum temperature (°C)**	**Winter Minimum temperature (°C)**	**Yearly Mean temperature (°C)**	**Growing degree days**
1995	767.7	18.3	−24.4	−2.53	183.13
1996	747.4	19.1	−23.6	−2.36	256.34
1997	763.5	20.5	−21.7	−2.01	389.18
1998	826.3	18.8	−27.5	−2.81	275.78
1999	737.4	20.0	−28.8	−2.46	259.30
2000	1069.4	18.4	−22.7	−1.71	242.38
2001	682.1	19.9	−27.6	−1.90	319.28

Yearly climate data for the 1994–1995 through 2000–2001 growing years, defined as beginning in October and extending through the winter, spring thaw, and summer growing seasons. Growing degree days were calculated from a threshold of 5 °C.

**Table 2:** Year-to-year differences in control plots.

**Response**	**group mean**	**Mean diff. from 1995**	**t-value**	**d.f.**	**p-value**
Total cover					
1995	46.375				
1999	48.250	1.875	0.297	21	0.77
2001	60.625	14.250	2.259	21	0.03
					
Shannon diversity index					
1995	1.399				
1999	1.163	−0.235	−1.345	21	0.19
2001	1.258	−0.141	−0.804	21	0.43
					
Total species richness					
1995	6.750				
1999	5.875	−0.875	−0.946	21	0.36
2001	6.750	0	0	21	1

A priori orthogonal contrasts comparing group means of total cover, species richness, and Shannon diversity in the eight control plots from 1995, the first year the plots were set up, to 1999 and 2001.
